# High prevalence of autoantibodies to RNA helicase A in Mexican patients with systemic lupus erythematosus

**DOI:** 10.1186/ar2905

**Published:** 2010-01-08

**Authors:** Monica Vázquez-Del Mercado, Claudia A Palafox-Sánchez, Jose F Muñoz-Valle, Gerardo Orozco-Barocio, Edith Oregon-Romero, Rosa E Navarro-Hernández, Mario Salazar-Páramo, Juan Armendariz-Borunda, Jorge I Gámez-Nava, Laura Gonzalez-Lopez, Jason YF Chan, Edward KL Chan, Minoru Satoh

**Affiliations:** 1 Departamento de Biología Molecular y Genómica, Instituto de Investigación en Reumatología y del Sistema Músculo Esquelético, Centro Universitario de Ciencias de la Salud, Universidad de Guadalajara, Sierra Mojada 950, Guadalajara, Jalisco, CP 44340, México; 2 División de Medicina Interna, Departamento de Reumatología, Hospital Civil 'Dr. Juan I. Menchaca', Salvador de Quevedo y Zubieta N° 750, CP 44340, Guadalajara, Jalisco, México; 3 Departamento de Inmunología y Reumatología del Hospital General de Occidente, Secretaría de Salud Jalisco, Av. Zoquipan 1050, CP 45100, Zapopan, Jalisco, México; 4 División de Investigación, Hospital de Especialidades, CMNO, IMSS, and Departamento de Fisiología, CUCS, Universidad de Guadalajara, Sierra Mojada 950, CP 44340, Guadalajara, Jalisco, México; 5 Departamento de Biología Molecular y Genómica, Instituto de Biología Molecular y Genómica, Centro Universitario de Ciencias de la Salud, Universidad de Guadalajara, Sierra Mojada 950, Guadalajara, Jalisco, CP 44340, México; 6 Servicio de Medicina-Interna-Reumatología, Hospital Regional 110, IMSS, Guadalajara, Jalisco, México; 7 Division of Rheumatology and Clinical Immunology, Department of Medicine, University of Florida, 1600 SW Archer Road, Gainesville, FL 32610-0424, USA; 8 Department of Oral Biology, University of Florida, 1600 SW Archer Road, Gainesville, FL 32610-0424, USA; 9 Department of Pathology, Immunology, and Laboratory Medicine, University of Florida, 1600 SW Archer Road, Gainesville, FL 32610-0221, USA

## Abstract

**Introduction:**

Autoantibodies to RNA helicase A (RHA) were reported as a new serological marker of systemic lupus erythematosus (SLE) associated with early stage of the disease. Anti-RHA and other autoantibodies in Mexican SLE patients and their correlation with clinical and immunological features were examined.

**Methods:**

Autoantibodies in sera from 62 Mexican SLE patients were tested by immunoprecipitation of ^35^S-labeled K562 cell extract and enzyme-linked immunosorbent assay (anti-U1RNP/Sm, ribosomal P, β2GPI, and dsDNA). Anti-RHA was screened based on the immunoprecipitation of the 140-kDa protein, the identity of which was verified by Western blot using rabbit anti-RHA serum. Clinical and immunological characteristics of anti-RHA-positive patients were analyzed.

**Results:**

Anti-RHA was detected in 23% (14/62) of patients, a prevalence higher than that of anti-Sm (13%, 8/62). Prevalence and levels of various autoantibodies were not clearly different between anti-RHA (+) vs. (-) cases, although there was a trend of higher levels of anti-RHA antibodies in patients without anti-U1RNP/Sm (*P *= 0.07). Both anti-RHA and -Sm were common in cases within one year of diagnosis; however, the prevalence and levels of anti-RHA in patients years after diagnosis did not reduce dramatically, unlike a previous report in American patients. This suggests that the high prevalence of anti-RHA in Mexican patients may be due to relatively stable production of anti-RHA.

**Conclusions:**

Anti-RHA was detected at high prevalence in Mexican SLE patients. Detection of anti-RHA in races in which anti-Sm is not common should be clinically useful. Racial difference in the clinical significance of anti-RHA should be clarified in future studies.

## Introduction

Systemic autoimmune rheumatic diseases such as systemic lupus erythematosus (SLE), scleroderma (systemic sclerosis), and polymyositis/dermatomyositis are serologically characterized by the production of autoantibodies to cellular constituents [1,2]. Although autoantibodies target various proteins, protein complexes, protein-nucleic acid complexes, and nucleic acids, selection of the target antigens is not a random event; rather, there can be a tight link between the specificity of autoantibodies each patient produces and the diagnosis or certain clinical symptoms. Some of the specificities are detected almost exclusively in patients with certain clinical diagnosis and considered pathognomonic. Anti-Sm and double-stranded DNA (dsDNA) antibodies are highly specific for the diagnosis of SLE and are included in the classification criteria [3]. While anti-dsDNA antibodies are found in approximately 70% of patients with SLE, their production fluctuates depending on the lupus activity and treatment they receive. Production of anti-Sm antibodies is generally considered more stable and is found in approximately 15% of patients with SLE; however, it is common in African-Americans and is low in prevalence in Caucasians [4]. Anti-ribosomal P and anti-PCNA (proliferating cell nuclear antigen) antibodies found in approximately 10% and approximately 2% of patients with SLE also are considered specific for SLE [1]. We have recently reported that, in addition to these classic markers, autoantibodies to RNA helicase A (RHA, also known as DNA helicase II), a 3'-5' dsDNA/RNA helicase [5] that belongs to the DExH superfamily of helicases, are a new serological marker of SLE [1,4]. In the previous report, the rates of prevalence of anti-RHA were 6% (8/133) in Caucasians, 2.9% (3/103) in African-Americans, and 12% (3/25) in the Latin population in the US. Another earlier report was also from the US [6]. Except for preliminary data suggesting that approximately 10% of Japanese patients with SLE are also positive [7], anti-RHA in other countries has not been reported. Anti-RHA is also unique in that it is associated with the early stage of the disease, typically within a year of diagnosis of SLE. However, the number in the Latin population was too small to analyze in the previous study [4]. In the present study, we determined the prevalence of anti-RHA and examined the clinical and immunological characteristics of anti-RHA-positive Mexican patients with SLE.

## Materials and methods

### Patients

Sixty-two consecutive patients with SLE from the Department of Rheumatology, Hospital General de Occidente, Zapopan, Jalisco, Mexico, were studied. All patients fulfilled the 1982 American College of Rheumatology (ACR) SLE classification criteria [3]. Mex-SLEDAI (Mexican Systemic Lupus Erythematosus Disease Activity Index) and Systemic Lupus International Collaborating Clinics/ACR Damage Indexes at the beginning of the study were evaluated [8,9]. Complete blood count, including lymphocyte count and serum rheumatoid factor (CELL-DYN 3500R; Abbott Diagnostics, Chicago, IL, USA), was determined in all subjects. Information on treatment of the day of sampling, including use of immunosuppressive drugs (azathioprine, methotrexate, and cyclophosphamide), chloroquine, and dose of steroid (milligrams of prednisone per day), was recorded. The protocol was approved by the institutional review board. This study meets and is in compliance with all ethical standards in medicine, and written informed consent was obtained from all patients according to the Declaration of Helsinki.

### Screening of autoantibodies in human sera by immunoprecipitation

Immunoprecipitation (IP) using ^35^S-methionine-labeled K562 cell extract to determine IgG class autoantibodies was performed using 8 μL of sera as described [10]. Specificities such as anti-U1RNP, Sm, ribosomal P, Ro, La, Ku, argonaute 2 (Ago2)/Su, and RNA polymerase II (RNAP II) were verified using previously described reference sera. Positive anti-U1RNP was defined based on the presence of the set of U1RNP proteins (A, B'/B, C, D1/D2/D3, E/F, and G). Since autoantibodies to U5RNP without anti-Sm are very rare [11], IP of the characteristic U5RNP 200-kDa proteins was used to define anti-Sm (which immunoprecipitates U2, U4-6, and U5 in addition to U1RNP) [10].

Anti-RHA was first screened based on IP of the 140-kDa protein. Selected sera were then re-run on 8% SDS-PAGE to verify that the mobility of the 140-kDa protein was the same as that of RHA immunoprecipitated by the reference sera. The identity of the 140-kDa protein as RHA was further confirmed by IP-Western blot (WB) as previously described [12]. Briefly, non-radiolabeled K562 cell extract from 5 × 10^6 ^cells was immunoprecipitated with 2 μL of serum that immunoprecipitated the 140-kDa protein in ^35^S-IP. Samples were run on 8% SDS-PAGE and transferred to a nitrocellulose filter. The filter was probed by rabbit anti-RHA antiserum (1:2,000, a gift from Jun-Qi Yang and Michael B Mathews, University of Medicine and Dentistry of New Jersey, Newark, NJ, USA) [13] followed by 1:2,000 horseradish peroxidase-labeled goat IgG F(ab')_2 _anti-rabbit IgG (γ-chain- and light-chain-specific; SouthernBiotech, Birmingham, AL, USA) and developed using SuperSignal West Pico Chemiluminescent Substrate (Pierce, Rockford, IL, USA).

### Quantification of anti-RHA antibody levels

Levels of anti-RHA were estimated and analyzed using the Storm Phosphorimager and images were obtained on a storage phosphor screen (Amersham Biosciences, now part of GE Healthcare, Little Chalfont, Buckinghamshire, UK) from 8% SDS-PAGE gels. The integrated density (that is, the sum of the values of the pixels in the image or selection; this is equivalent to the product of the area and the mean gray value) [14] of the RHA on a phosphorimage was calculated using ImageJ software (National Institutes of Health, Bethesda, MD, USA).

### Enzyme-linked immunosorbent assay

Sera were tested for IgG anti-U1RNP/Sm, ribosomal P (P peptide), dsDNA, and β2 glycoprotein I (β2GPI) (a gift from Junichi Kaburaki, Tokyo Electric Power Company Hospital, Tokyo, Japan) antibodies by enzyme-linked immunosorbent assay (ELISA) as described [15,16]. P peptide was COOH-terminal 22 amino acids of human P0 protein [17]. dsDNA was purified using S1 nuclease as described [15]. Anti-U1RNP/Sm antigen-capture ELISA was performed as described [16]. Briefly, microtiter plates (Immobilizer Amino™; Nalge Nunc, Rochester, NY, USA) were coated with 3 μg/mL mouse monoclonal antibodies (mAbs) 2.73 (IgG2a, anti-U1-70k) [10]. The left half of the plate was incubated with K562 cell lysate (50 μL/well, 4 × 10^7^/mL), and the right half was incubated with the blocking buffer as control. After the plate was washed, an identical set of samples and serially diluted standard serum (1:500 to serial 1:5 dilutions) were added to the left and right halves (control for reactivity against mouse IgG) of the plate. Serum samples were tested at 1:500 and 1:2,500 dilutions, and data from the latter were used for the analysis. Plates were washed with Tris-buffered saline/Tween20, incubated with alkaline phosphatase-conjugated mouse mAbs to human IgG (Sigma-Aldrich, St. Louis, MO, USA) (1:1,000 dilution), and developed. The optical density (OD) of 405 nm of wells was converted into units based on the standard curve, and the units of the corresponding right half (without U1RNP/Sm antigens) were subtracted from the left half (with antigens) using SoftMax Pro 4.3 software (Molecular Devices Corporation, Sunnyvale, CA, USA) [15]. For the detection of anti-P peptide, dsDNA, and β2GPI, microtiter plates were incubated with 1 to 3 μg/mL of the appropriate antigens, and ELISA was performed as described previously using 1:500 diluted sera. ODs were converted into units as described using the appropriate standard [15].

### Statistical analysis

All statistical analysis was performed using Prism 5.0 for Macintosh (GraphPad Software, Inc., San Diego, CA, USA). The Fisher exact test and the Mann-Whitney test or Student *t *test were used to analyze prevalence and levels, respectively, of autoantibodies and other data.

## Results

A previous study suggested that anti-RHA was associated with an early stage of SLE within a few years of diagnosis [4]. Consistent with this observation, the levels of anti-RHA dramatically decreased over time. Studies in mouse models suggested that the environment for the production of anti-RHA was distinct from that of anti-small nuclear ribonucleoproteins (anti-snRNPs) or anti-Su (Ago2) [12,18]. Opposite kinetics of production of anti-RHA versus anti-snRNPs or anti-ribosomal P, observed in some human cases, may be reminiscent of these observations [4,7]. Thus, we examined whether these findings in the previous human study performed in an American (mainly Caucasian) population [4] also apply to Mexican patients with SLE and whether anti-RHA and anti-snRNPs have a negative correlation.

### Screening of anti-RHA antibodies

**Figure 1:**
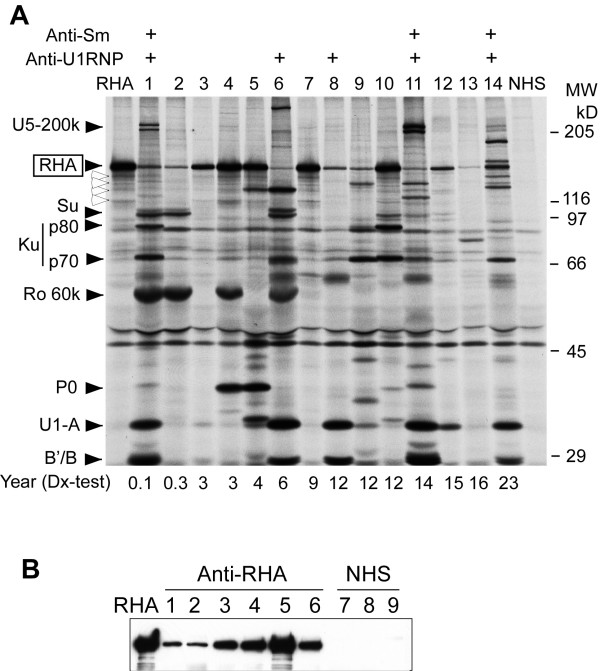
**Analysis of autoantibodies to RNA helicase A (RHA)**. **(a) **Immunoprecipitation using anti-RHA-positive sera. Immunoprecipitation of ^35^S-methionine-labeled K562 cell extract by anti-RHA-positive sera from Mexican patients with systemic lupus erythematosus (SLE) (n = 14), anti-RHA prototype serum (lane RHA), and a normal human serum (NHS) is shown. Number of years between initial diagnosis and anti-RHA test of each patient is indicated below the lanes. Positions of RHA, UsnRNP components A, B'/B, U5-200k doublet, Ku (p70 and p80), Ro 60k, and ribosomal P P0, and molecular weight (MW) are indicated. Positivity of anti-Sm and U1RNP is indicated at the top. White arrowheads indicate major degradation products of RHA. **(b) **Immunoprecipitation and Western blot confirmation of anti-RHA. K562 cell extract was immunoprecipitated by sera positive for the 140-kDa protein that co-migrated with RHA. Identity of the 140-kDa protein as RHA was validated by Western blot using a rabbit anti-RHA serum. Lane RHA, anti-RHA prototype serum; lanes 1 to 6, anti-RHA-positive sera screened by immunoprecipitation; lanes 7 to 9, NHS.

Anti-RHA antibodies were screened based on the IP of the 140-kDa protein using ^35^S-methionine-labeled K562 cell extract as described [4]. Sera selected were run along with the prototype sera to verify the identical mobility (Figure 1a). Several sera with strong reactivities showing typical degradation patterns (white arrowheads, see lanes RHA, 4, 5, 7, and 10) were clearly anti-RHA; however, it is possible that there are other proteins that co-migrate with RHA. Thus, IP-WB using rabbit anti-RHA serum was also performed to verify the identity of proteins immunoprecipitated by each serum (Figure 1b). All 14 sera initially selected by IP were positive by IP-WB, confirming that the sera indeed had anti-RHA. Clinical and immunological characteristics of these 14 patients were compared with those of anti-RHA-negative patients.

### Prevalence of anti-RHA antibodies and stage of the disease

Anti-RHA was found in 23% (14/62) in this cohort of Mexican patients, a prevalence that was greater than that of anti-Sm (13%) (Table 1) and much higher than that of anti-RHA (6%) reported in American patients [4], whereas the prevalence of other specificities did not seem to be different from other reports in SLE.

**Table 1:** Frequency of autoantibodies in Mexican patients with systemic lupus erythematosus

	Total	Anti-RHA (+)	Anti-RHA (-)
Number of patients	62	14	48
RHA	23% (14/62)		
U1RNP	29% (18/62)	36% (5/14)	27% (13/48)
Sm	13% (8/62)	21% (3/14)	10% (5/48)
Anti-Sm (+) among anti-U1RNP (+)	44% (8/18)	60% (3/5)	38% (5/13)
Ribosomal P	8% (5/62)	14% (2/14)	6% (3/48)
Ro	39% (24/62)	29% (4/14)	42% (20/48)
La	8% (5/62)	0% (0/14)	10% (5/48)
Ku	6% (4/62)	14% (2/14)	4% (2/48)
Su	24% (15/62)	29% (4/14)	23% (11/48)
RNAP II	10% (6/62)	14% (2/14)	8% (4/48)

Not significant between anti-RHA (+) and (-) groups for all specificities by Fisher exact test. RHA, RNA helicase A; RNAP II, RNA polymerase II.

**Figure 2:**
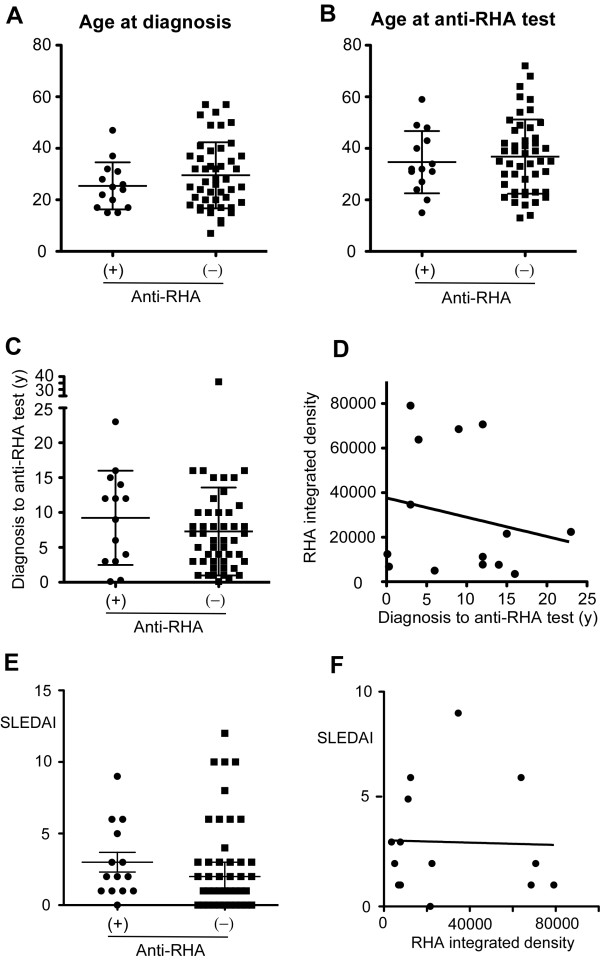
**Age at diagnosis, age at anti-RNA helicase A (anti-RHA) test, and years between diagnosis and anti-RHA test**. Demographic data of anti-RHA-positive (n = 14) and -negative (n = 48) systemic lupus erythematosus (SLE) patients were compared. **(a) **Age at diagnosis. **(b) **Age at anti-RHA test. **(c) **Years from diagnosis to anti-RHA test. **(d) **Years from diagnosis to anti-RHA test versus levels of anti-RHA. **(e) **Systemic Lupus Erythematosus Disease Activity Index (SLEDAI) in anti-RHA (+) versus (-) patients. **(f) **Correlation of SLEDAI and levels of anti-RHA. Anti-RHA levels were measured as integrated density of RHA protein band using phosphorimager as described in Materials and methods. y, years.

The production of anti-RHA was associated with an early stage of SLE in American patients [4]. To examine whether this applies to Mexican patients, the prevalence of anti-RHA was compared in groups classified based on the years between diagnosis and screening test (Table 2). Two of four patients within a year of diagnosis had anti-RHA; however, 9 out of 14 cases of anti-RHA were after 5 years of diagnosis, suggesting that anti-RHA does not disappear in Mexican patients with SLE. This is in striking contrast to the Caucasian population [7]. Also, the prevalence of anti-RHA was the same as or higher than that of anti-Sm in patients even more than 5 or 10 years after diagnosis. These results were quite different from the pattern reported in the previous American study [4]. Distributions of age at diagnosis, age at anti-RHA test, and years between diagnosis and anti-RHA test were compared between anti-RHA-positive (left in each panel) and -negative (right) patients (Figure 2a, b, and 2c, respectively). All showed no clear difference between anti-RHA (+) and (-) groups.

**Table 2:** Frequency of anti-RHA and years from diagnosis

Years between diagnosis and test	Number of patients	Anti-RHA (n = 14)	Anti-Sm (n = 8)
0 to less than 1	4	50% (2/4)	75% (3/4)
1 to less than 2	5	0% (0/5)	0% (0/5)
2 to less than 5	15	20% (3/15)	0% (0/15)
5 to less than 10	17	12% (2/17)	12% (2/17)
10 or more	21	33% (7/21)	14% (3/21)

RHA, RNA helicase A.

### Levels of anti-RHA antibodies and stage of the disease and SLEDAI

Next, whether the levels of anti-RHA in patients years after diagnosis are lower than those of patients with early-disease status was evaluated by comparing the intensity of RHA IP and years after diagnosis (Figure 2d). Although the linear regression analysis suggested a negative association between these two, it was not statistically significant. It appeared that the samples with very strong anti-RHA antibodies and the ones with weak anti-RHA were both scattered throughout the different stages of the disease, suggesting that anti-RHA levels do not decrease dramatically, unlike in American patients in the previous report [4].

Detailed information on SLE classification criteria was available from 12 anti-RHA (+) and 42 anti-RHA (-) patients. The prevalence of each SLE criteria was compared between anti-RHA (+) and (-) groups to examine whether anti-RHA (+) SLE had unique clinical features. The prevalence of all criteria items appeared to be similar between groups (data not shown). Whether anti-RHA (+) SLE had different disease activity was evaluated by comparing SLEDAI between anti-RHA (+) and (-) patients, but no clear difference was found (Figure 2e). No correlation between the levels of anti-RHA antibodies and disease activity was observed (Figure 2f).

### Levels of various autoantibodies and anti-RHA antibodies

**Figure 3:**
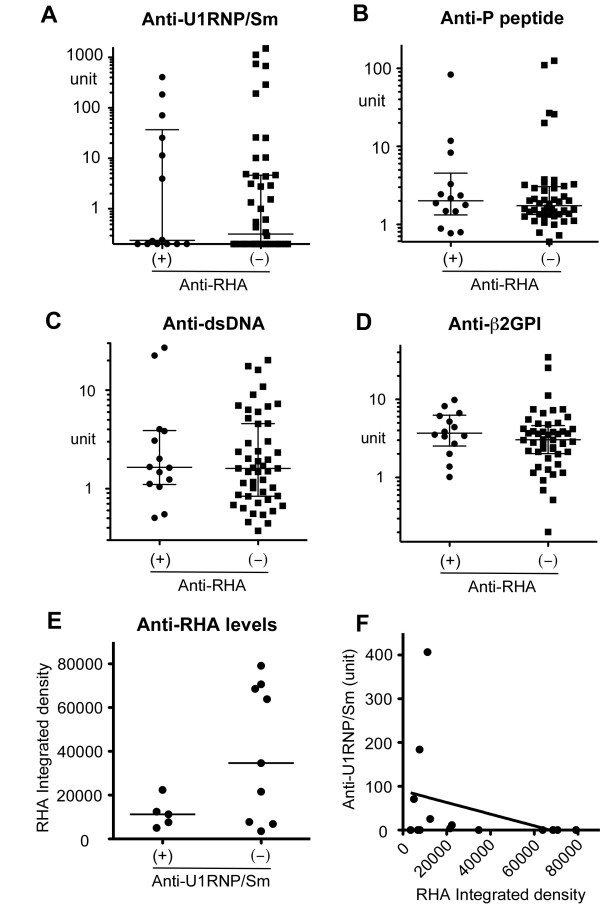
**Levels of anti-RNA helicase A (anti-RHA) versus other autoantibodies**. IgG anti-U1RNP/Sm, ribosomal P (P peptide), double-stranded DNA (dsDNA), and β2 glycoprotein I (β2GPI) were determined by enzyme-linked immunosorbent assay. Serum dilutions used were 1:2,500 for anti-U1RNP/Sm and 1:500 for all others. Anti-RHA levels were semiquantified from immunoprecipitation using phosphorimager. **(a) **Anti-U1RNP/Sm antibodies. **(b) **Anti-P peptide antibodies. **(c) **Anti-dsDNA. **(d) **Anti-β2GPI. **(e) **Anti-RHA levels in anti-U1RNP/Sm-positive versus -negative sera. **(f) **Correlation of anti-RHA versus anti-U1RNP/Sm.

The prevalence of coexisting autoantibodies was not significantly different between anti-RHA-positive and -negative patients (Table 1). Levels of anti-U1RNP/Sm, -ribosomal P (P peptide), dsDNA, and β2GPI antibodies by ELISA were compared between anti-RHA-positive and -negative cases (Figure 3a-d); however, the difference was not apparent for any of these specificities.

The levels of anti-RHA quantified by phosphorimager and those of anti-U1RNP/Sm by ELISA were compared. Although the subjects with high levels of anti-RHA may be more common in the anti-U1RNP/Sm-negative group, the difference did not reach statistical significance (*P *= 0.07 by Student *t *test) (Figure 3e). None of the anti-U1RNP/Sm (+) cases (0/5) had an anti-RHA integrated density of greater than 30,000 versus 56% (5/9) in the anti-U1RNP/Sm (-) group (*P *= 0.086 by Fisher exact test). There was a trend of negative correlation between levels of anti-RHA and anti-U1RNP/Sm; however, it was not statistically significant (Figure 3f).

## Discussion

Anti-Sm antibody is a well-established serological marker of SLE and is one of the minor criteria under immunological disorders of the SLE classification criteria [3]. However, it is found in only approximately 15% of SLE patients and in particular it is uncommon in Caucasians [19]. Thus, an additional serological marker specific for SLE should be clinically useful. Anti-RHA has recently been reported as a new serological marker of SLE in the US [4]. The rates of prevalence in the previous study were 6% in the general SLE population, approximately 6% in Caucasians and African-Americans, and 12% in the Latin population; however, only 3 out of 25 Latin patients were positive. The present study showed a much higher prevalence of anti-RHA: 23% in Mexican patients with SLE. Although anti-RHA appears specific for SLE in the previous study regardless of the race, the number of Latin patients was relatively small [4]. Disease specificity of anti-RHA in other ethnicities, including Mexicans, will need to be established in future studies. In contrast to previous data [4], the presence of anti-RHA was not skewed toward patients in an early stage of the disease in the current study (Table 1). A comparison of years between diagnosis and anti-RHA test versus levels of anti-RHA (Figure 2d) did not show a clear negative correlation, suggesting a relatively stable production of anti-RHA over time in Mexican patients. There are several potential explanations for the discrepancy. One possibility is the racial difference. The previous study on anti-RHA-positive SLE included 8 Caucasians but only 3 African-Americans and 3 Latin patients, indicating that the data were much affected by the characteristics of Caucasian anti-RHA-positive SLE patients. A recent analysis of the same study population showed that the majority of Caucasian patients with anti-RHA were at an early stage of SLE; 5 out of 8 anti-RHA patients were within one year of diagnosis [7]. However, this may not be the case for other races, a circumstance that may potentially explain the difference.

A second difference is a distribution of different stages of SLE patients for reasons that are not clear; the previous American study had a large percentage of patients in an early stage of SLE [4]. In the previous study, the percentage of patients within 1 or 2 years into the disease was nearly twice that of the present study (13% versus 7%, 27% versus 15%, within 1 year and 2 years, respectively). Although the reduction of anti-RHA levels did not appear to be a simple result of immunosuppressive therapy, current and historical treatment may also have effects on the data mentioned above. These points should be addressed in future studies with a large number of patients from different ethnicities.

A hydrocarbon oil pristane induces autoantibodies to snRNPs, ribosomal P, and Ago2/Su in normal mice [20,21]; however, it inhibits the production of anti-RHA antibodies in two strains of mice that spontaneously produce anti-RHA: NZB/W F1 [12] and CBA/n (x-linked immunodeficiency) [18]. In the former, pristane induced anti-snRNP and anti-Ago2/Su antibodies [12]. Human anti-RHA-positive cases that newly developed anti-snRNPs or -ribosomal P antibodies while their anti-RHA levels drop significantly [4] appear to be reminiscent of the observations in animal models. Thus, it seems reasonable to hypothesize that the environment or stage of the disease that is preferable for the production of anti-RHA is not ideal for the production of other specificities. Although the difference was not statistically significant (*P *= 0.07, Student *t *test) (Figure 3e), five cases with the highest levels of anti-RHA were in the anti-snRNP-negative group. These may be the cases that are going to 'switch' the specificities from anti-RHA to anti-snRNPs later, like the previously described cases [4]. Factors that are responsible for the production of anti-RHA or switching from anti-RHA to anti-snRNPs in humans are not known; however, in animal models of pristane injection, which switches autoantibody specificity from anti-RHA to anti-snRNP production, type I interferon (I-IFN) [22,23] and Th1 cytokine shifting are induced [12]. Thus, switching of autoantibody specificities in humans may also involve changes in cytokine balance, in particular Th1 cytokine or I-IFN production, or Toll-like receptor (TLR) 7 stimulation since the production of pristane-induced anti-snRNPs in animal models is dependent on I-IFN [24] and TLR7 [25]. Environmental factors such as viral infection may trigger this type of change via stimulation of TLRs or via a TLR-independent mechanism of I-IFN induction [26]. Although the production of lupus autoantibodies is generally considered an event prior to typical clinical manifestation [27], it is of interest that 10% to 15% of autoantibodies develop after the diagnosis, in particular within a year of diagnosis [1]. The development of anti-snRNPs after steroid treatment reported in clinically MCTD (mixed connective tissue disease) patients [28,29] is interesting when considering a role of treatment as a trigger to change the environment of autoantibody production, although differentiating the natural course from induction by steroids is virtually impossible. The identification of mechanisms of induction and regulation of various autoantibodies may help develop a strategy of therapeutic regulation of autoantibody production.

## Conclusions

The present study reports a high prevalence of anti-RHA in Mexican patients with SLE. The detection of SLE-specific autoantibodies in addition to anti-Sm should be clinically helpful, in particular in the population of patients with a low prevalence of anti-Sm. Patients with high levels of anti-RHA appear to be more common among anti-snRNP-negative patients. A strong association of anti-RHA with an early stage of SLE was not apparent in the Mexican population, possibly due to the relatively stable production of anti-RHA over time. Possible differences in clinical significance of anti-RHA in different races should be clarified in future studies.

## Abbreviations

β2GPI: β2 glycoprotein I; ACR: American College of Rheumatology; Ago2: argonaute 2; dsDNA: double-stranded DNA; ELISA: enzyme-linked immunosorbent assay; I-IFN: type I interferon; IP: immunoprecipitation; mAb: monoclonal antibody; OD: optical density; RHA: RNA helicase A; SLE: systemic lupus erythematosus; SLEDAI: Systemic Lupus Erythematosus Disease Activity Index; snRNP: small nuclear ribonucleoprotein; TLR: Toll-like receptor; WB: Western blot.

## Competing interests

The authors declare that they have no competing interests.

## Authors' contributions

MVDM and MS designed the study, performed experiments, and wrote the manuscript. CAPS, JFMV, GOB, EOR, RENH, MSP, JAB, JIGN, and LGL helped perform experiments and interpret data. JYFC and EKLC helped perform experiments, interpret data, and write the manuscript. All authors read and approved the final manuscript.
